# CT‐Based Body Composition and Frailty as Predictors of Survival Among Older Adults With Gastrointestinal Malignancies

**DOI:** 10.1002/jcsm.13664

**Published:** 2024-12-23

**Authors:** Smith Giri, Christian Harmon, Daniel Hess, Elizabeth M. Cespedes Feliciano, Ijeamaka Anyene Fumagalli, Bette Caan, Leon Lenchik, Karteek Popuri, Vincent Chow, Mirza Faisal Beg, Smita Bhatia, Grant R. Williams

**Affiliations:** ^1^ Institute for Cancer Outcomes and Survivorship University of Alabama at Birmingham Birmingham Alabama USA; ^2^ Department of Medicine, Division of Hematology and Oncology University of Alabama at Birmingham Birmingham Alabama USA; ^3^ Department of Medicine University of Alabama at Birmingham Birmingham Alabama USA; ^4^ Division of Research Kaiser Permanente, Northern California Oakland California USA; ^5^ Department of Radiology Wake Forest University School of Medicine Winston‐Salem North Carolina USA; ^6^ Department of Computer Science Memorial University of Newfoundland St John's Newfoundland and Labrador Canada; ^7^ School of Engineering Science Simon Fraser University Burnaby British Columbia Canada

**Keywords:** aging, frailty, geriatric oncology, myosteatosis, sarcopenia

## Abstract

**Background:**

Older adults with cancer are at an increased risk of treatment related toxicities and early death. Routinely collected clinico‐demographic characteristics inadequately explain this increased risk limiting accurate prognostication. Prior studies have suggested that altered body composition and frailty are independently associated with worse survival among older adults with cancer; however, their combined influence remains unclear.

**Methods:**

We used data from a single‐institution prospective cohort study of older adults (≥ 60 years) who underwent geriatric assessment (GA) at the time of initial consultation with a medical oncologist from September 2017 to December 2020 and available baseline abdominal computed tomography within 60 days of GA. Using multi‐slice CT images from T12 to L5 level, we assessed volumetric measures of skeletal muscle (SMV), visceral adipose tissue (VATV), subcutaneous adipose tissue (SATV) and averaged skeletal muscle density (SMD), computing sex‐specific *z* for each measure. Frailty was measured using a 44‐item frailty index using the deficit accumulation approach. Primary outcome of interest was overall survival (OS) defined as time from GA to death or last follow up. We used multivariable Cox regression model to study the independent association between the above four body composition measurements and OS adjusted for baseline confounders and frailty.

**Results:**

We included 459 patients with a mean age of 69.7 ± 7.5 years, 60% males and 77% non‐Hispanic Whites. Most had colorectal (27%) or pancreatic cancer (20%) and 48% had stage IV disease. Over a median follow up of 39.4 months, 209 patients (46%) died. In multivariable Cox regression models adjusted for age, sex, race, cancer type, cancer stage and frailty, skeletal muscle volume (HR 0.74; 95% CI 0.58–0.96; *p* = 0.02, per 1 SD increment) was independently associated with OS. The addition of body composition variables to baseline clinico‐demographic variables and frailty led to a slightly improved model discrimination.

**Conclusions:**

SMV is independently associated with OS among older adults with newly diagnosed gastrointestinal cancers. Capturing body composition measurements in oncology practice may provide additional prognostic information for older adults with cancer above and beyond what is captured in routine clinical assessment including frailty.

## 1 Introduction

By 2040, an estimated 70% of all new cancer diagnoses will occur among adults aged ≥ 65 years [1]. As compared with their younger counterparts, older adults are at an increased risk of treatment related toxicities and excess mortality. Accurate prediction of the risk of early mortality among older adults with cancer is essential to guide clinical decision‐making and for early institution of palliative therapies to optimize quality of life and minimize aggressive end of life care. This is particularly true for more aggressive cancers such as gastrointestinal (GI) malignancies that comprise a large proportion of older adults and are associated with high disease related morbidity and mortality [2].

Prior studies have shown that chronologic age and clinician assessed performance status alone are insufficient in explaining the heterogeneity of treatment outcomes evident in older adults with cancer [3, 4]. A geriatric assessment (GA) systematically examines multiple domains of aging‐related health and is recommended in the routine management of all older adults with cancer [5, 6, 7]. Whereas time and resource constraints have limited broader uptake of GA in routine oncologic practice [8], the development of pragmatic GAs is poised to overcome such barriers [9, 10]. Furthermore, changes in body composition including progressive loss in muscle mass and/or gain in adiposity are associated with aging process as well as underlying drivers and biomarkers of physiologic frailty. Measuring skeletal muscle and adipose tissue compartments may thus help characterize the variability and heterogeneity in the aging process and aid in predicting individual morbidity and mortality [11]. The introduction of automated tools to capture such information using routinely available computed tomography imaging obtained during clinical care have made such information readily available for clinicians [12].

Available prognostic models among older adults with cancer have traditionally relied heavily on chronologic age, baseline clinico‐demographic factors and more recently GA variables, with limited use of body composition measurements [13, 14, 15, 16]. Although both frailty and altered body composition measurements [such as low muscle mass (commonly referred to as sarcopenia) or excess visceral adiposity] are known predictors of survival among older adults with cancer, very few studies have incorporated both GA data and body composition variables within the same analyses, and the incremental value of body composition measurements above and beyond frailty assessment remains unknown. To that end, the goal of the current study was to determine whether body composition measures (muscle mass, muscle density, subcutaneous and visceral adipose tissue) is independently associated with survival among older adults (age ≥ 60 years) with newly diagnosed GI malignancies.

## 2 Methods

### 2.1 Study Design, Setting and Participants

We used data from the Cancer and Aging Resilience Evaluation (CARE) Registry, an ongoing prospective registry of older adults treated at the University of Alabama at Birmingham (UAB) Hospital and Clinics with a specific emphasis on GI malignancies. Older adults (age ≥ 60 years) with a GI malignancy seen for consultation at UAB complete a pragmatic self‐reported GA as part of routine clinical care [17]. Given the uncertainty of the appropriate age cut‐off for ‘older patients’ and the poor correlation of age and impairments in geriatric assessment [4], the age of ≥ 60 years was chosen as eligibility for the GA tool in this cohort. To be eligible for this study, participants were required to have (a) known diagnosis of GI malignancy presenting for an initial visit at UAB between September 2017 and August 2021, (b) enrolled in CARE registry, (c) underwent GA within 90 days of diagnosis, (d) complete frailty and anthropometric data and (e) available computed tomography (CT) images for body composition analysis within 60 days of GA. All patients consented for participation in this registry which was approved by the UAB Institutional Review Board (IRB‐300000092).

### 2.2 Study Measures

#### 2.2.1 Exposure: Body Composition

We included four body composition measurements of interest: skeletal muscle (SM), skeletal muscle density (SMD), subcutaneous adipose tissue (SAT) and visceral adipose tissue (VAT). For all four tissue types, volumetric measurements were obtained across multi‐slice scans spanning T12‐L5 vertebrae using an automated segmentation platform [Data Analysis Facilitation Suite (DAFS), Voronoi Health Analytics, Inc.] [18, 19]. Volumetric indices of muscle and adipose tissue measured using DAFS has been shown to have excellent agreement with manual segmentation (Dice coefficient ranging from 0.96 to 0.98) and is independently associated with worse survival in a cohort of adults with patients with colorectal cancer [20, 21]. Meanwhile, SMD was computed using mean radiodensity of skeletal muscle measured in terms of Hounsfield units (HU). All automated segmentations were independently reviewed by two investigators (CH & SG) for quality assurance. Agreement rates on tissue quantifications between the two reviewers were measured using inter‐rater reliability coefficient.

#### 2.2.2 Frailty

Frailty was measured using a deficit accumulation method using 44‐variables from the CARE GA as previously described [22]. These variables comprised various GA domains ranging from falls, activities of daily living, instrumental activities of daily living, health related quality of life, nutrition, psychosocial health, co‐morbid conditions and polypharmacy. Of the 44 items, we required at least 30 variables to be non‐missing to compute a valid frailty index (Data S1). Patients were categorized as robust (0–0.2), pre‐frail (0.2–0.35) and frail (> 0.35), as previously described [23]. We have previously shown that among older adults with GI cancers, CARE‐Frailty Index is associated with risk of overall mortality, treatment‐related toxicity and functional decline [24, 25].

#### 2.2.3 Outcome

Our outcome of interest was overall survival (OS) defined as time between date of the GA to death from any cause. Ascertainment of mortality was be done by linkage to Accurint database [26], which uses death information from Social Security Administration records, obituaries and state death records; we supplemented mortality data with manual review of UAB Electronic Health Records. All patients were followed through 2 December 2022.

#### 2.2.4 Other Variables

We obtained demographic characteristics including age at time of GA evaluation, self‐reported sex (male and female) and self‐reported race and ethnicity (White and others). Measured height and weight data were abstracted from clinical records within 60 days of study enrolment. We abstracted clinical characteristics including cancer type (colorectal, pancreatic, hepatobiliary, gastroesophageal and others) and cancer stage (stage I–IV), and chemotherapy regimen intensity from review of electronic health records. We used the number of chemotherapeutic agents used (none vs. single vs. doublet vs. three or more) as a measure of regimen intensity.

### 2.3 Statistical Analysis

We described the baseline characteristics of the study cohort by survival status, reporting mean (SD) age median score (along with interquartile range) of frailty index and body composition variables, and frequency and count of categorical variables. We compared the distribution of baseline covariates by survival status using appropriate bivariate statistics (i.e., *t*‐test for continuous variables and Pearson's chi‐squared statistic for categorical variables). We measured the correlation between the four body composition variables using Pearson's correlation and visualized using scatterplot matrix.

We reported the median follow up time of the entire cohort using reverse Kaplan–Meier method [27]. We characterized the overall survival characteristics of the study population using product limit methods. Subsequently, we developed Cox proportional hazard models to study the association between each body composition measure and all‐cause mortality. We administratively censored patients who were still alive at end of study period (December 2 2022) and ensured that proportionality assumption were met for all the key variables prior to analysis using the Kolmogorov type supremum test using 1000 simulated patients [28]. No automated variable selection method were used, and the initial set of demographic and clinical covariates (age, sex, race/ethnicity, cancer type and cancer stage, regimen intensity and frailty) to be included in the base model were selected a priori based on prior clinical experience and literature review. We then forced all four body composition measurements in the base model to study the independent association between body composition measurements on OS. Measured height was included as a covariate in the model in order to account for differences in volumetric body composition as a function of body size. The added predictive value of body composition measurements on OS was evaluated using likelihood ratio tests, improvement in model discrimination (Harrel's C‐statistic and Uno's C statistic) and Akaike information criteria. All hypothesis testing was two sided, and the level of significance was set at 0.05. We used STATA, version 16 (StataCorp LLC, College Station, TX, USA) and SAS version 9.4 (SAS Institute Inc., Cary, NC, USA) for all statistical analysis.

## 3 Results

Of the 1009 consecutive patients with newly diagnosed GI malignancy present for an initial medical oncology visit at our centre between September 2017 and August 2021, 459 had available data on frailty and body composition and were selected for further analysis (Data S1). Patients in the final analytic sample vs. those who were excluded were similar in age, or sex distribution but were more likely to be White and less likely to have pancreatic cancer and stage IV disease (Data S1). Our study cohort underwent GA and body composition assessment within a median of 22 days (range 0–90 days) and 18 days (8–30 days) from the date of diagnosis. The overall cohort had a mean age of 69.7 ± 7.5 years, with 60% males, and 77% White. Colorectal (27%) and pancreatic cancer (20%) comprised almost half of our sample, and 48% had stage IV disease. Most patients (62%) were receiving first line of therapy. The overall distribution by body composition variables was as follows: skeletal muscle volume (mean 2149 ± 708 cm^3^), skeletal muscle density (mean 40.2 ± 11.1 HU), subcutaneous adipose tissue (mean 3514.6 ± 195 cm^3^) and visceral adipose tissue (2924.4 ± 1918.6 cm^3^) (Table 1). We found a moderate positive correlation between skeletal muscle and visceral adipose tissue volumes (*r* = 0.58), as well as subcutaneous adipose tissue and visceral adipose tissue volumes (*r* = 0.43) (Data S1).

**TABLE 1:** Baseline demographics and clinical characteristics of the study population.

Variable	Overall (*N* = 459)
Age, mean ± SD	70 (7)
Sex	
Male	255 (56%)
Female	204 (44%)
Race	
White	335 (73%)
Others	120 (26%)
Missing	4 (1%)
Cancer type	
Colorectal	148 (32%)
Pancreatic	135 (29%)
Other GI	176 (38%)
Cancer stage	
Stage I	46 (10%)
Stage II	91 (20%)
Stage III	133 (29%)
Stage IV	188 (41%)
Unknown	1 (0%)
Frailty score, median (IQR)	0.27 (0.16, 0.40)
Frailty category	
Robust	154 (34%)
Pre‐frail	150 (33%)
Frail	155 (34%)
SM volume in cm^3^, mean ± SD	2078 (689)
SM density in HU	40 (12)
SAT volume in cm^3^, mean ± SD	3528 (1920)
VAT volume in cm^3^, mean ± SD	2893 (1902)

Abbreviations: GI, gastrointestinal; IQR, interquartile range; SAT, subcutaneous adipose tissue; SD, standard deviation; SM, skeletal muscle; VAT, visceral adipose tissue.

### 3.1 Association Between Frailty and Overall Survival

**FIGURE 1:**
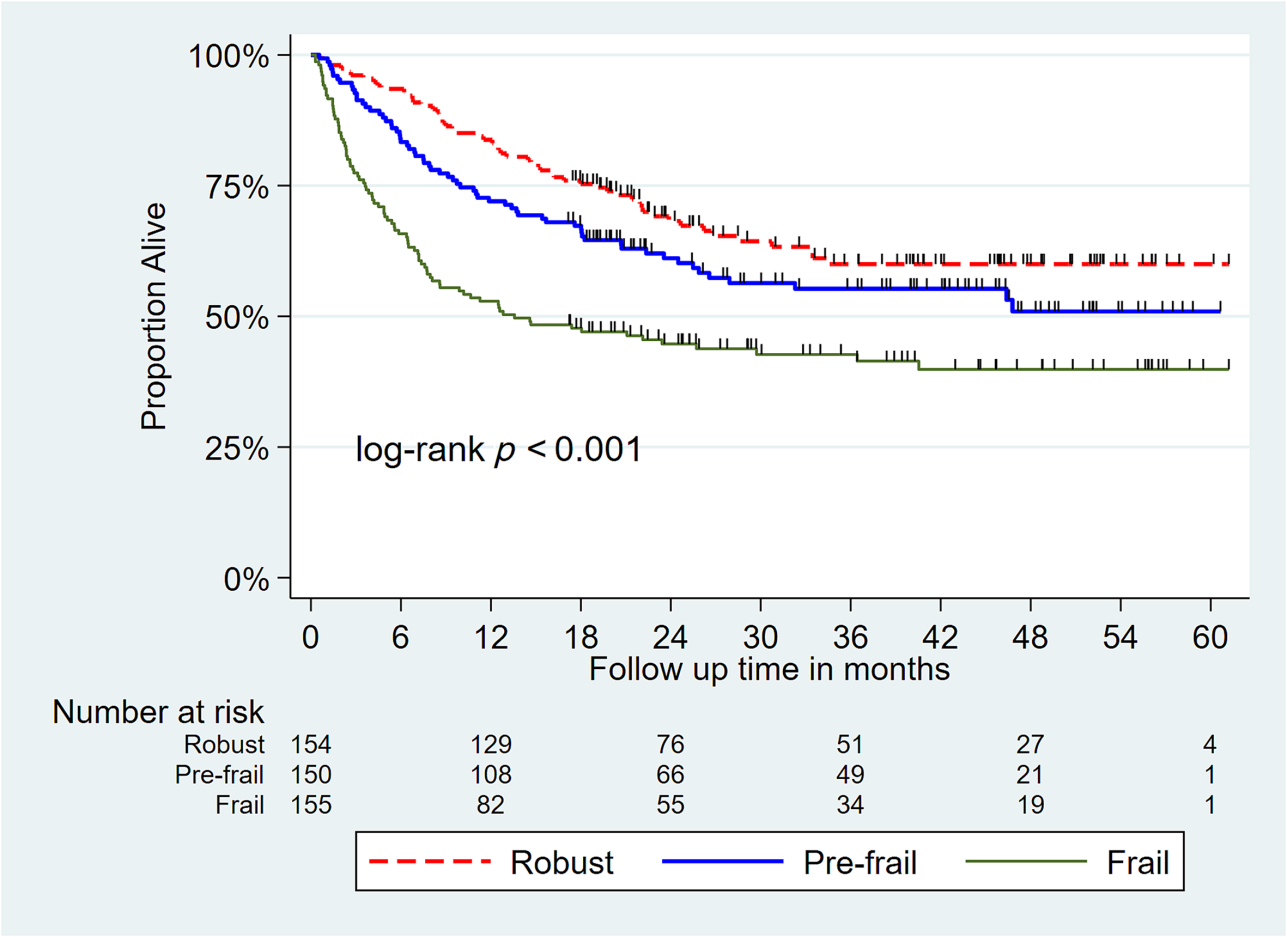
Comparison of survival distribution between different frailty classes. As compared with robust patients, those who were pre‐frail or frail had significantly worse survival estimates (log rank *p* value < 0.001).

Over a median follow up of 39.4 (range 0.3–61.1) months, a total of 209 patients (45.5%) died. The median OS of the overall cohort was not reached, but the 1 y and 3 year OS was 70% and 53%, respectively. Of these, 174 (38%) patients died within 1 year of study enrolment. The median frailty score was 0.27 (interquartile range 0.16–0.40); 33% (*N* = 150) and 31% (*N* = 155) were characterized as pre‐frail and frail, respectively. The 3‐year OS rate for robust, pre‐frail and frail patients was 60%, 55% and 43%, respectively (log rank *p* value < 0.001) (Figure 1).

### 3.2 Association Between Body Composition and Overall Survival

We ensured that the proportional hazards assumption was met for all key covariates in the model. In our multivariable model, we found that skeletal muscle volume were independently associated with the risk of all‐cause mortality after adjusting for confounders including the frailty score. Specifically, we found that each 1 SD increase in skeletal muscle volume was associated with a 26% reduction in the risk of all‐cause mortality (95% CI 0.58–0.96; *p* value 0.02). There was a trend towards increased risk of mortality with increasing VAT (hazards ratio, HR 1.25; 95% CI 0.99–1.56; *p* value 0.051 per 1 SD increase), although this was not statistically significant (Table 2). Addition of body composition variables to the base model (including frailty) led to slightly improved model discrimination in terms of Harrel's C‐statistic (0.68 to 0.71), Uno's C‐statistic (0.65–0.67) and model fit using Akaike information criteria (Table 3).

**TABLE 2:** Association between body composition measures and all‐cause mortality among older adults with gastrointestinal malignancy (*N* = 454).^a^

Variable	Hazards ratio (95% CI)	*p*
SM volume, per SD increase	0.74 (0.58–0.96)	0.02
SMD, per SD increase	0.92 (0.78–1.10)	0.37
SAT volume, per SD increase	0.87 (0.72–1.04)	0.13
VAT volume, per SD increase	1.25 (0.99–1.55)	0.05
Frailty Score, per 0.1 unit increase	1.23 (1.13–1.34)	< 0.001

*Note:* Standard deviation values for SM 689.1 cm^3^, SMD 11.67 Hounsfield units, SAT 1919.9 cm^3^, and VAT 1902 cm^3^.

Abbreviations: CI, confidence interval; SD, standard deviation; SAT, subcutaneous adipose tissue; SM, skeletal muscle; SMD, skeletal muscle density; VAT, visceral adipose tissue.

^a^ Adjusted for age, sex, race/ethnicity, measured height (continuous), cancer type and cancer stage.

**TABLE 3:** Evaluation of Cox regression model performance with and without inclusion of body composition measurements.

Model performance	Base model including frailty	Model with body composition variables
Harrel's C‐statistic (± 95% CI)	0.68 (0.65–0.71)	0.71 (0.67–0.74)
Uno's C‐statistic (± 95% CI)^a^	0.65 (0.62–0.69)	0.67 (0.64–0.71)
AIC	2363.82	2346.1
BIC	2405	2387.3

Abbreviations: AIC, Akaike information criteria; BIC, Bayesian information criteria.

^a^ Uno's C statistic was computed over a time horizon of 36 months.

## 4 Discussion

Our study suggests that body composition measurements, specifically volumetric measurements of skeletal muscle predict survival among older adults with gastrointestinal cancer independent of demographic/clinical variables and frailty. These findings highlight the importance of obtaining precise body composition measurements to better understand the treatment outcome heterogeneity among older adults with cancer thus providing valuable information to patients and clinicians to help guide appropriate care.

Despite the increasing recognition of using geriatric assessment and frailty to better delineate the functional age of older adults with cancer, such assessments are not routinely done in oncology practice. A recent survey by the American Society of Clinical Oncology suggested that < 20% of providers routinely performed a GA in clinical care with lack of time and lack of resources cited as two most common barriers to implementation [8]. At our institution, we have recently pioneered the development of a pragmatic patient‐reported GA and have shown that such assessments can be successfully integrated in routine oncology practice [17]. Moreover, we have shown that a frailty index, captured using such a patient reported GA, can provide meaningful clinical information to clinicians by identifying patients at risk of adverse outcomes [22]. The current study further expands the utility of our frailty index, that is, prediction of older adults at risk of 1 year mortality.

In addition to frailty, our findings support the clinical utility of quantifying skeletal muscle mass among older adults with cancer [11]. Prior studies have shown that low skeletal muscle mass is associated with worse progression free and overall survival for both solid tumours and hematologic malignancies [29]. However, to our knowledge, no study have looked at the incremental benefit of using skeletal muscle measures beyond what is captured by GA and frailty. To that end, our findings suggest that frailty and body composition assessment can provide complementary information that can help identify older adults at risk of early mortality during cancer therapy.

Whereas it can be argued that both frailty and skeletal muscle mass measurements are not routinely available in day to day practice, there are active ongoing efforts to do so. The American Society of Clinical Oncology has recently developed a pragmatic GA with a goal of streamlining frailty assessment in oncology care [5]. Others have developed frailty scales using patient reported questionnaires [30] or using information captured in the electronic medical records (EMR) [31]. Similarly, there has been a parallel development in automated and software platforms which can streamline the process of extracting body composition variables from routinely obtained clinical imaging [12, 20]. Taken together, these efforts are poised to make frailty and body composition information readily available in oncology practice.

Our study has several limitations. First most patients had GI malignancies and were recruited from a single site in the United States in the Deep South. It is conceivable that our findings may not be readily generalizable to other malignancies or populations. Further, the frailest patients with the highest risk of death may not have enrolled in our study as we observed that our participants were less likely to have stage IV disease or have pancreatic cancer than those who did not complete a geriatric assessment. While not all patients had archived CT scans available for body composition assessment, we did not see a systematic difference in frailty or baseline characteristics between those who did or did not have available imaging. Lastly, we found that the four body composition measures were moderately correlated with each other which may have limited our ability to identify independent effects of a particular body composition variable on outcomes.

In conclusion, our study demonstrates that body composition, specifically skeletal muscle mass and visceral adipose tissue, is independently associated with all‐cause mortality among older adults with cancer independent of demographic/clinical risk factors and frailty. In addition to validating our findings in diverse settings, future efforts should attempt to uncover the potential mechanisms of these associations to develop mitigating interventions and improve outcomes in this population.

## Ethics Statement

This study was approved by the UAB Institutional Review Board (IRB‐300000092).

## Conflicts of Interest

SG: honoraria: CareVive and OncLive; research funding: Carevive Systems, Pack Health and Sanofi. The other authors declare no conflicts of interest.

## Supporting information


**Figure S1.** Flowchart outlining the study cohort selection process. Out of 1009 patients newly diagnosed GI cancers presenting during the study period, 459 were included in the final analytic sample. Of the 220 patients whose CT scans were missing, 69 (31.4%) had no scans available in our image repository, whereas the remaining 151 (68.6%) had images that were obtained outside of the 60 day window from the time of the GA.
**Table S1.** Baseline Characteristics between study participants vs. non‐participants. GI, gastrointestinal.
**Figure S2.** Scatter plot matrix visualizing and quantifying the correlation between the four body composition variables. Skeletal Muscle volume (SMV) and Visceral Adipose Tissue (VAT) were moderate positively correlated with each other (pearson correlation r = 0.58). Similarly, Subcutaneous Adipose Tissue (SAT) and VAT were moderate positively correlated with each other (r = 0.43). A weak positive correlation was seen between SMV and SAT (r = 0.15) and a weak negative correlation was seen between skeletal muscle density (SMD) and SAT (r = −0.28). Asterisks indicate that the correlation coefficient was significantly different from 0 at the 5% level of significance.
