# Supplementary material for: CT‐Based Body Composition and Frailty as Predictors of Survival Among Older Adults With Gastrointestinal Malignancies
Source: J Cachexia Sarcopenia Muscle. 2024 Dec 23;16(1):e13664. doi: 10.1002/jcsm.13664 (PMC11744297; doi:10.1002/jcsm.13664)
Supplement: Supplementary file 1 — Figure S1. Flowchart outlining the study cohort selection process. Out of 1009 patients newly diagnosed GI cancers presenting during the study period, 459 were included in the final analytic sample. Of the 220 patients whose CT scans were missing, 69 (31.4%) had no scans available in our image repository, whereas the remaining 151 (68.6%) had images that were obtained outside of the 60 day window from the time of the GA. Table S1. Baseline Characteristics between study participants vs. non‐participants. GI, gastrointestinal. Figure S2. Scatter plot matrix visualizing and quantifying the correlation between the four body composition variables. Skeletal Muscle volume (SMV) and Visceral Adipose Tissue (VAT) were moderate positively correlated with each other (pearson correlation r = 0.58). Similarly, Subcutaneous Adipose Tissue (SAT) and VAT were moderate positively correlated with each other (r = 0.43). A weak positive correlation was seen between SMV and SAT (r = 0.15) and a weak negative correlation was seen between skeletal muscle density (SMD) and SAT (r = −0.28). Asterisks indicate that the correlation coefficient was significantly different from 0 at the 5% level of significance. [file JCSM-16-e13664-s001.docx]

**CT-based Body Composition and Frailty as predictors of survival among Older Adults with Gastrointestinal Malignancies.**

**Supplement:**

**Table of Contents.**

1. Flow chart illustrating study cohort selection …………………………………..Page 1
2. Baseline characteristics between study participants vs non-participants.......Page 2
3. Scatter plot matrix showing the correlation between the four body composition variables ……………………………………………………………………………………….Page 3
4. Construction of the CARE Frailty Index …………………………………,,,,,,,,..Page 4

Patients undergoing GA after 90 days of diagnosis

(N=81)

Patients with missing data on Frailty (N=29), height (N=20) and body composition (220)

Patients undergoing GA within 90 days of diagnosis

N=459

Patients consented for enrollment in CARE Registry and underwent baseline GA

N=540

Patients consented for enrollment in CARE Registry and underwent baseline GA

N=816

Consecutive patients with newly diagnosed GI cancers presenting for an initial medical oncology visit between 9/2017-08/2021

N=1009

Patients not consented

- missed (58)

- Not consented due to COVID-19 (18)

-Refused (117)

**Figure S1:** Flowchart outlining the study cohort selection process. Out of 1009 patients newly diagnosed GI cancers presenting during the study period, 459 were included in the final analytic sample. Of the 220 patients whose CT scans were missing, 69 (31.4%) had no scans available in our image repository, whereas the remaining 151 (68.6%) had images that were obtained outside of the 60 day window from the time of the GA.

| Variable | Overall  (N=1009) | Participants  (N=459) | Non-participants  (N=550) | P value |
| --- | --- | --- | --- | --- |
| **Age, mean +/- SD** | 69 (7) | 69 (7) | 70 (7) | 0.08 |
| **Sex**  **- Male**  **- Female** | 567 (56%)  442 (44%) | 255 (56%)  204 (44%) | 312 (57%)  238 (43%) | 0.71 |
| **Race**  **- White**  **- Others**  **- Missing** | 697 (69%)  278 (28%)  34 (3%) | 335 (73%)  120 (26%)  4 (1%) | 362 (66%)  158 (29%)  30 (5%) | <0.001 |
| **Cancer Type**  **- Colorectal**  **- Pancreatic**  **- Other GI** | 323 (32%)  261 (26%)  425 (42%) | 148 (32%)  135 (29%)  176 (38%) | 175 (32%)  126 (23%)  249 (45%) | 0.03 |
| **Cancer Stage**  **- Stage I**  **- Stage II/III**  **- Stage IV**  **- Unknown** | 75 (7%) 475 (47%)  446 (44%)  13 (1%) | 46 (10%)  224 (49%) 188 (41%)  1 (0.2%) | 29 (5%) 251 (46%)  258 (47%)  12 (2%) | <0.001 |

**Table S1: Baseline Characteristics between study participants vs non-participants**

GI, gastrointestinal.

**Figure S2:** Scatter plot matrix visualizing and quantifying the correlation between the four body composition variables. Skeletal Muscle volume (SMV) and Visceral Adipose Tissue (VAT) were moderate positively correlated with each other (pearson correlation r=0.58). Similarly, Subcutaneous Adipose Tissue (SAT) and VAT were moderate positively correlated with each other (r=0.43). A weak positive correlation was seen between SMV and SAT (r=0.15) and a weak negative correlation was seen between skeletal muscle density (SMD) and SAT (r=-0.28). Asterisks indicate that the correlation coefficient was significantly different from 0 at the 5% level of significance.
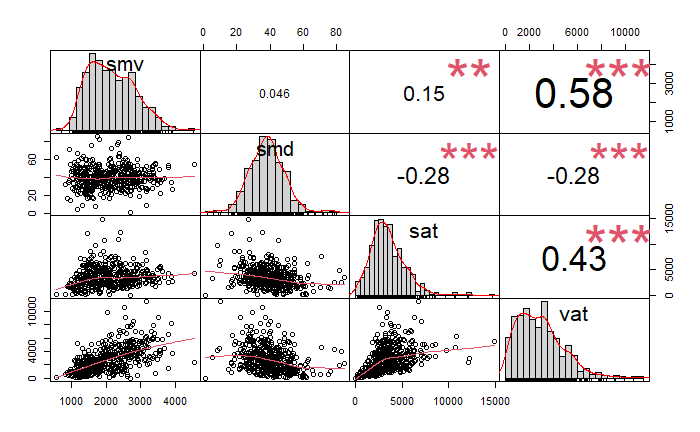


**Appendix 1: Construction of CARE Frailty Index:**

We constructed a frailty index (hereafter known as the CARE Frailty Index) using the deficit accumulation approach originally described by Rockwood et al^12^, and following the standard procedures outlined by Searle et al^13^. Similar methods have been used by Guerard et al^14^ and Cohen et al^15^ to construct frailty indices that have been shown to be predictive of chemotherapy toxicity and drug discontinuation^15^ as well as all-cause mortality^14^ among older adults with cancer. We selected 44 GA variables from the CARE survey, each of which captured a health deficit, and recoded responses as ‘0’ for absence of the deficit and ‘1’ for presence of the deficit. For variables that included a single intermediate response (e.g. ‘sometimes’ or ‘maybe’), we used an additional value of ‘0.5’. We combined the 44 individual scores into an aggregate frailty score reflecting the overall proportion of deficits (range 0-1), and then categorized patients as robust (0-0.2), pre-frail (0.2-0.35) or frail (>0.35), as previously described.^13^ In case of missing data, we required responses to at least 30 items to construct a valid frailty index. An index constructed with at least 30 variables has been previously shown to be sufficiently accurate for predicting adverse outcomes among older adults.^16^ The 44 variables used for construction of CARE-frailty index are as below.

1. Falls ≥1, 1 point
2. Walk one block = limited a lot , 1 point
3. IADL mobility (unable to/ with some help) , 1 point
4. IADL shopping (unable to/ with some help) , 1 point
5. IADL meal prepare ( unable to/ with some help) , 1 point
6. IADL housework ( unable to/ with some help) , 1 point
7. IADL medication ( unable to/ with some help) , 1 point
8. IADL money ( unable to/ with some help) , 1 point
9. ADL get in and out of bed ( unable to/ with some help) , 1 point
10. ADL dress ( unable to/ with some help) , 1 point
11. ADL bath ( unable to/ with some help) , 1 point
12. Global health, good =0.5 point, fair/poor = 1 point
13. Global quality of life, good =0.5 point, fair/poor = 1 point
14. Global physical health, good =0.5 point, fair/poor = 1 point
15. Global mental health, good =0.5 point, fair/poor = 1 point
16. Global satisfaction with social activities and relationship, good =0.5 point, fair/poor = 1 point
17. Global everyday activities, moderately=0.5 point, a little/not at all = 1 point
18. Global anxious/depression, sometimes =0.5 point, often/always = 1 point
19. Global fatigue, moderate =0.5 point, severe/very severe = 1 point
20. Global pain, pain level 4-6 =0.5 point, pain level 7-10 = 1 point
21. Global social activities and roles, good =0.5 point, fair/poor = 1 point
22. Weight loss 3 months or 6 months’ weight loss >=5%, 1 point
23. Food intake less than usual, 1 point
24. Activities and function (self-rated activity) ≥2 (in bed or chair less than half the day/ able to do little activity / Pretty much bedridden) , 1 point
25. Anxiety PROMIS T score >60, 1 point
26. Depression PROMIS T score >60, 1 point
27. Impaired Cognition, PROMIS T score <40, 1 point
28. Number of daily medication ≥9, 1 point
29. Social activity interference, Some of the time=0.5 point, Most/ All of the time=1 point

**Comorbidities:**

1. Eyesight Fair/Poor/totally blind, 1 point
2. Hearing fair/Poor/Totally Deaf, 1 point
3. Other Cancers or leukemia, 1 point
4. Arthritis or rheumatism, 1 point
5. Glaucoma, 1 point
6. Emphysema or chronic bronchitis, 1 point
7. High blood pressure, 1 point
8. Heart disease, 1 point
9. Circulation trouble in arms or legs, 1 point
10. Diabetes, 1 point
11. Stomach or intestinal disorders, 1 point
12. Osteoporosis, 1 point
13. Chronic liver or kidney disease, 1 point
14. Stroke, 1 point
15. Depression, 1 point
